# A Case of Iron Deficiency Anemia with Co-existing Hb Fontainebleau

**DOI:** 10.4084/MJHID.2014.051

**Published:** 2014-07-01

**Authors:** Abhishek Purohit, Mukul Aggarwal, Roshan B Colah, Anita H Nadkarni, Hara P Pati

**Affiliations:** 1 Department of Hematology, All India Institute of Medical Sciences, New Delhi, India; 2 Institute of Immunohaematology, Haematogenetics. Mumbai, Maharashtra, India

## Abstract

Hb Fontainebleau is a rare alpha chain variant in the Indian population which generates an unknown peak on hemoglobin HPLC study and does cause diagnostic difficulty to those who are not acquainted with this entity. We present a case of Hb Fontainebleau, an eighteen year old patient who presented with symptoms related to anemia to our department and unknown peak observed in HPLC plots lead us to family study and molecular characterization for this case.

## Introduction

Alpha-thalassaemia, one of the most common human genetic abnormalities known, are inherited as an autosomal recessive disorder. They have clinical phenotype varying from almost asymptomatic to a lethal haemolytic anemia and can occur due to deletional or non-deletional mutations. Non-deletional alpha thalassemias are relatively rare, and cause more severe anemia clinically. Hb Fontainebleau is one of the rarely reported clinically asymptomatic α chain variants.^1^ It shows a characteristic retention time on hemoglobin HPLC (High-performance liquid chromatography) which may cause diagnostic dilemma if one is unaware of its pattern. We are reporting a case of Hb Fontainebleau, who attended our hematology clinic for symptoms related to anemia. This patient represent the sixth case of this alpha chain variant reported worldwide.

## Case Presentation

An eighteen year old Punjabi male born to a non-consanguineous marriage presented to hematology department with two year history of weakness and easy fatigability. His past and family history was not significant. Physical examination revealed pallor only. There was no icterus, lymphadenopathy or hepatosplenomegaly. Systemic examination did not reveal any abnormality.

**Figure 1:**
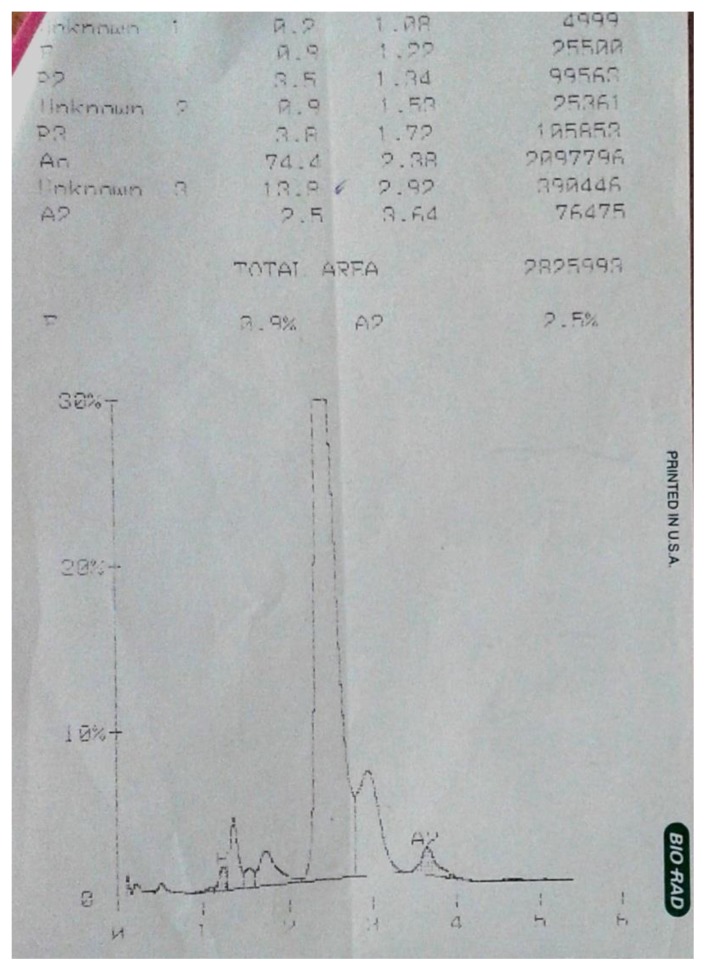
High-performance liquid chromatography chromatogram showing the unknown peak at retention time 2.92 minutes.

Laboratory investigation revealed hemoglobin 7.2 g/dL, total leucocyte count 6.01 × 10^9^/L with normal differential count and platelet count 141 × 10^9^/L. Peripheral smear examination revealed microcytic hypochromic red cells with mean corpuscular volume (MCV) 68.9 fL, mean corpuscular hemoglobin (MCH) 15.8 pg and mean corpuscular hemoglobin concentration (MCHC) being 22.9 g/dL and red cell count 4.56 × 10^12^/L. His serum ferritin was 10 ng/ml; serum iron was 12 μg/dL, total iron binding capacity was 589 μg/dL, unbound iron binding capacity was 587 μg/dL and transferrin saturation was 2.03%. HPLC analysis (BioRad, Beta thalassaemia short program) on the patient’s blood revealed 13.8% of an unknown hemoglobin with retention time of 2.92 minutes which appeared as a hump in the peak adjoining Hb A (Figure 1).

The other hemoglobins on HPLC were 77.6% of Hb A, 2.3% of Hb A2 and 1.2% of Hb F. Cellulose acetate electrophoresis (pH 8.9) did not show any abnormal band. HPLC of the parental samples was performed which revealed the presence of a similar 11.6% unknown hemoglobin with retention time 2.87 minutes in the mother. Her hemoglobin was 10.3 g/dL with MCV 81.7 fL, MCH 25.1 pg and MCHC 307 g/L red cell count 4.10 × 10^12^/L. Father had hemoglobin of 12.0 g/dL with MCV 59.0 fL, MCH 18.6 pg and MCHC 315 g/L red cell count 6.46 × 10^12^/L. His HPLC analysis revealed raised Hb A2 (5.4%) suggesting heterozygous beta thalassaemia.

**Figure 2:**
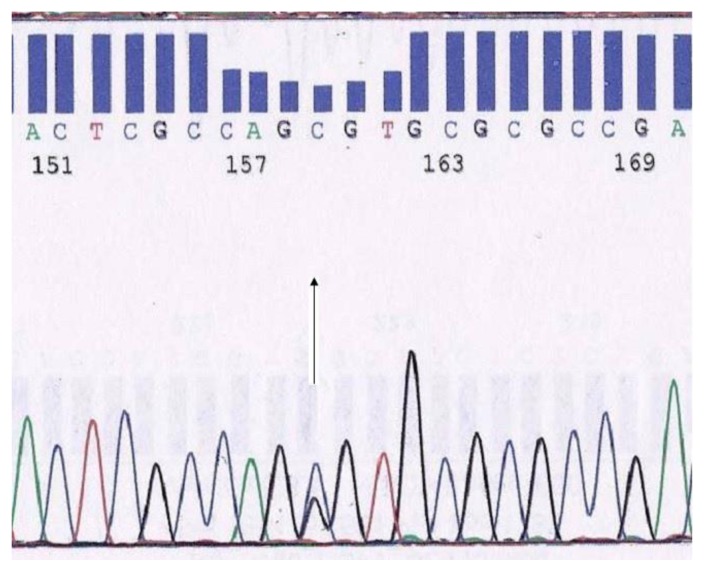
Electropherogram of the alpha gene showing hemoglobin Fontainebleau [Alpha 21(B2) α2Ala>Pro].

Molecular characterization was done by automated DNA sequencing on the ABI Prism 310 DNA sequencer (Applied Systems, Foster City, CA, USA.) showing presence of a heterozygous G>C substitution at codon 21 (alpha 2 globin gene) leading to the substitution of alanine to proline at the beginning of the beta helix in the alpha chain corresponding to Hb Fontainebleau in the index case, his mother and two sisters (Figure 2). The father was heterozygous for beta thalassaemia with the CD 8/9(+G) mutation. The patient was treated with oral iron supplements due to low ferritin levels and responded well to therapy with relief of symptoms and increase in hemoglobin on the subsequent evaluation.

## Discussion

Mutations that cause alpha thalassemia can be deletional or non-deletional types. Non-deletional alpha thalassemias are relatively rare, result in more severe anaemia and are because of a variety of mutations including punctual substitutions and small insertions/deletions.^2^ The majority of these mutations are found to involve the upstream α2 globin gene proximal to the erythroid-specific regulatory region called multispecies conserved sequences (MCS).^3^

Many mutations have been described affecting mRNA processing, mRNA translation, and α-globin stability. Hb Fontainebleau [alpha 21 (B2) Ala> Pro, HBA2 : c 64G>C] is an alpha chain variant characterized by an alanine→proline substitution at codon 21 with a GCT>CCT change at the DNA level, this proline residue is located at the beginning of the beta helix.^4^ Literature search reveals only five reported cases of Hb Fontainebleau worldwide, including two cases from India.^5^–^8^

The first reported case of Hb Fontainebleau was that of an adolescent female of Italian origin, who had severe anemia. However, this severity was explained by the co-existing membrane defect, spherocytosis with Hb Fontainebleau.

This hemoglobin variant has electrophoretic properties identical to those of Hb A with the exception of isoelectrofocusing in which it migrates like Hb A1c. The introduction of a prolyl residue at the beginning of the B helix in the alpha chain does not lead to a change in the stability or oxygen binding properties of the hemoglobin molecule.^4^

With identification of a second case it was observed that although slightly unstable, this variant was expressed at 28–29% of the total and was caused by a heterozygous mutation in the alpha2 gene. This second reported case was an adult Iraqi male, living in New Zealand.^5^ The third case was seen during thalassemia screening in the Greek Cypriot population in Cyprus.^6^ Upadhye et al, in their newborn screening for sickle cell disorders, identified a baby with Hb Fontainebleau in a compound heterozygous state with Hb S and the baby had anemia at birth (Hb 11.4 g/dL), however had no cyanosis, icterus or need for transfusion.^7^ The second case from India was a 35 year old lady, a case of secondary infertility who presented for routine antenatal screening program for thalassemia and had normal hematological indices.^8^ The present case was different from other reported cases from India as it was not part of a screening programme, but rather presented due to his moderately severe anemia. However causal relationship of anemia with Hb Fontainebleau cannot be established as he responded well to iron supplements and achieved near normal hemoglobin level. Further, his mother and both the sisters were asymptomatic.

## Conclusions

Hb Fontainebleau is a rare alpha chain variant in the Indian population. It generates an unknown peak on HPLC and can cause diagnostic difficulty for those who are not acquainted with this entity. However this produces no symptoms of anemia so a patient presenting with anemia and Hb Fontainebleau should be investigated for other causes of anemia and counseled properly.
